# Hypothyroidism Exacerbates Thrombophilia in Female Rats Fed with a High Fat Diet

**DOI:** 10.3390/ijms160715776

**Published:** 2015-07-10

**Authors:** Harald Mangge, Florian Prüller, Sieglinde Zelzer, Herwig Ainödhofer, Sabine Pailer, Petra Kieslinger, Johannes Haybaeck, Barbara Obermayer-Pietsch, Gerhard Cvirn, Hans-Jürgen Gruber

**Affiliations:** 1 Clinical Institute for Medical and Chemical Laboratory Diagnostics, Medical University Graz, 8036 Graz, Austria; E-Mails: Florian.Prueller@klinikum-graz.at (F.P.); sieglinde.zelzer@klinikum-graz.at (S.Z.); Herwig.Ainoedhofer@klinikum-graz.at (H.A.); sabine.pailer@klinikum-graz.at (S.P.); Petra.Kieslinger@klinikum-graz.at (P.K.); hans.gruber@medunigraz.at (H.-J.G.); 2 Department of Pathology, Medical University of Graz, 8036 Graz, Austria; E-Mail: johannes.haybaeck@medunigraz.at; 3 Division of Endocrinology and Metabolism, Medical University Graz, 8036 Graz, Austria; E-Mail: barbara.obermayer@medunigraz.at; 4 Physiological Chemistry, Medical University of Graz, 8036 Graz, Austria; E-Mail: gerhard.cvirn@medunigraz.at

**Keywords:** endogenous thrombin potential (ETP), hyperthyroidism, hypothyroidism, fat feeding, Sprague Dawley rats

## Abstract

Clotting abnormalities are discussed both in the context with thyroid dysfunctions and obesity caused by a high fat diet*.* This study aimed to investigate the impact of hypo-, or hyperthyroidism on the endogenous thrombin potential (ETP), a master indicator of clotting activation, on Sprague Dawley rats fed a normal or high fat diet. Female Sprague Dawley rats (*n* = 66) were grouped into normal diet (ND; *n* = 30) and high-fat diet (HFD; *n* = 36) groups and subdivided into controls, hypothyroid and hyperthyroid groups, induced through propylthiouracil or triiodothyronine (T3) treatment, respectively. After 12 weeks of treatment ETP, body weight and food intake were analyzed. Successfully induced thyroid dysfunction was shown by T3 levels, both under normal and high fat diet. Thyroid dysfunction was accompanied by changes in calorie intake and body weight. In detail, compared to euthyroid controls, hypothyroid rats showed significantly increased—and hyperthyroid animals significantly decreased—ETP levels. High fat diet potentiated these effects in both directions. In summary, we are the first to show that hypothyroidism and high fat diet potentiate the thrombotic capacity of the clotting system in Sprague Dawley rats. This effect may be relevant for cardiovascular disease where thyroid function is poorly understood as a pathological contributor in the context of clotting activity and obesogenic nutrition.

## 1. Introduction

Although thyroid hormones have been associated with increased cardiovascular risk and atherosclerosis [1,2], knowledge about this is inconsistent. Hypothyroid individuals, even those with subclinical disease, have been shown to have an impaired endothelial function, left ventricular systolic and diastolic dysfunction, dyslipidemia with elevated total cholesterol, low-density lipoprotein-cholesterol, and a decreased high-density lipoprotein-cholesterol [2]. Moreover, systemic low grade inflammation, increased homocysteine, and arterial stiffness are under debate [3]. Although considered occasionally as a pathological contributor, the coagulation system remains underinvestigated in the context of thyroid function and atherosclerosis. Hence, the existing data are contradictory and range from a hypercoagulable influence of hyperthyroidism, e.g., in cardiovascular patients [4,5,6,7] to the opposite, *i.e.*, a prothrombotic role of hypothyroidism [8].

We have recently shown that an obesogenic lifestyle significantly enhances ETP as early as in childhood [9] and may indicate an increased future risk for cardiovascular disease (CVD) in these individuals. Other studies confirmed a significant influence of diet on endogenous thrombin generation [10]. On the other hand, an increased ETP was seen to be associated with a better survival rate in adult, middle aged patients with overt CVD after myocardial infarction [11]. Hence, the exact role of this global coagulation parameter remains to be clarified—especially in connection with metabolic cofactors such as obesity and/or thyroid function. Not least, the so-called obesity paradox [12] may be causative for the apparently contradictory findings shown so far.

We hypothesize that both hypo- and hyperthyroidism are associated with altered ETP levels which may contribute to the generation of CVD. A high fat diet may well simulate the additional influence of an obesogenic lifestyle on these mechanisms. Thus, we aim to investigate the influence of thyroid dysfunctions on ETP under normal and high fat diet in Sprague Dawley rats.

## 2. Results

Successful induction of thyroid dysfunction was determined via T3 showing strongly increased total T3 levels T3 levels up to 2- to 3-fold in hyperthyroid groups and markedly decreased levels in hypothyroid groups, both under normal and high fat diet (HFD), Table 1. Analyses of body weight changes and food consumption revealed that, compared to appropriate controls, hyperthyroid rats showed body weight changes between 12% and 19%, and increased food consumption both regarding food intake and calorie intake as shown in Table 1. These effects are seen under normal as well as under HFD. In contrast, induction of hypothyroidism reduced body weights and food consumption compared to appropriate controls. In detail, under normal diet, up to −13% reduction of body weight, and about −50% of food intake were seen, which resulted in a decreased calorie uptake of −47%. These effects are also seen under HFD. To analyze the effects of high fat diet and thyroid dysfunctions lipid profiles and clotting parameters were determined.

**Table 1:** Characteristics of female Sprague Dawley (SD) rats after 12-week treatment.

Parameters	Normal Diet Group (*n* = 30)			High Fat Diet Group (*n* = 27)		
	Control (*n* = 10)	Hypothyroid (*n* = 10)	Hyperthyroid (*n* = 10)	Control (*n* = 10)	Hypothyroid (*n* = 10)	Hyperthyroid (*n* = 7)
Body weight change (g)	36 ± 24	−35 ± 8 ***	31 ± 7	56 ± 24	−42 ± 16 ***	49 ± 17 ^††^
Body weight change (%)	14 ± 10	−13 ± 2 ***	12 ± 3	21 ± 9	−15 ± 5 ***	19 ± 7
Food intake/rate/day (g)	19	10	30	13	8	18
Calorie intake/rat/day (kcal)	49	26	80	61	38	87
Total T3 (pg/mL)	417 ± 85	269 ± 74 **	951 ± 454 **	450 ± 58	446 ± 176 ^†^	1708 ± 1447
Triglyceride (mg/dL)	62 ± 7.7	46 ± 4.5 ***	61 ± 36.5	50 ± 12.4 º	32 ± 6.1 ^†††^	88 ± 14.1
Free fatty acids (mmol/L)	1.10 ± 0.17	0.70 ± 0.18 **	1.01 ± 0.29	0.83 ± 0.11 ºº	0.53 ± 0.14	1.20 ± 0.51
HDL (mg/dL)	44 ± 5.4	39 ± 6.0	41 ± 7.4	44 ± 5.2	66 ± 4.6 ^†††^	40 ± 8.7
Cholesterol (mg/dL)	86 ± 13	87 ± 14	69 ± 17 *	81 ± 14	185 ± 23 ^†††^	64 ± 13
Free cholesterol (mg/dL)	24 ± 3.6	26 ± 5.1	19 ± 6.5 *	20 ± 5.2	59 ± 8.5 ^†††^	18 ± 6.7
ETP (%)	61.3 ± 2.9	82.8 ± 4.5 ***	49.5 ± 5.6 ***	69.3 ± 9.7	129 ± 10.4 ^†††^	62.9 ± 3.1
PZ INR	0.83 ± 0.02	0.9 ± 0.2	0.83 ± 0.07	0.96 ± 0.3	0.83 ± 0.05	0.81 ± 0.05
APTT (s)	24 ± 8	38 ± 10 **	21 ± 2	31.2 ± 8	52 ± 5 ^†††^	26.2 ± 6
Fibrinogen (g/L)	0.87 ± 0.1	1.39 ± 0.4 ***	0.9 ± 0.1	0.94 ± 0.08	1.74 ± 0.4 ^†††^	1.25 ± 0.5
Antithrombin (%)	124 ± 4	117 ± 15	116 ± 28	108 ± 21	91 ± 23	106 ± 15

Data are presented as means ± standard deviations * *p* < 0.05, ** *p* < 0.01, *** *p* < 0.001 compared to appropriate control; º *p* < 0.05, ºº *p* < 0.01, compared to normal diet control group; ^†^ *p* < 0.05, ^††^ *p* < 0.01, ^†††^ *p* < 0.001 compared to appropriate normal diet group.

As shown in Table 1, triglycerides and free fatty acids were significantly decreased in the hypothyroid rats compared to controls in both diet groups. Interestingly, HDL levels were significantly increased in the HFD hypothyroid group (Table 1). No statistical significant differences were seen in the hyperthyroid groups concerning triglycerides, free fatty acids and HDL.

Analyses of cholesterol and free cholesterol showed significantly decreased levels between hyperthyroid rats and controls under normal diet. Hypothyroid rats under HFD had significantly increased levels (Table 1).

**Figure 1:**
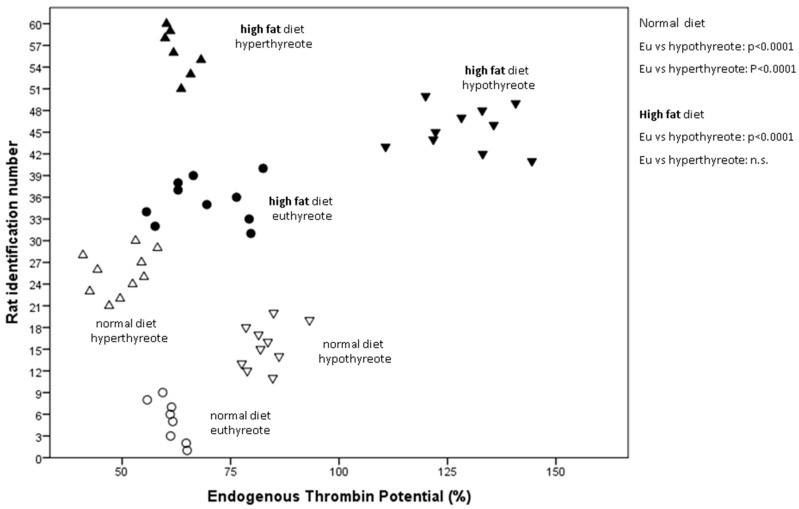
Levels of endogenous thrombin potential within the different treatment groups of hypo-, normo-, and hyperthyreote rats. Rats with hypothyreosis who receive a high fat diet show by far the highest endogenous thrombin potential levels. Hypothyroid rats under normal diet have also significantly increased endogenous thrombin potential levels compared to the euthyreote control group.

As shown in Figure 1 and Table 1, in the normal diet group, hypothyroid rats had significantly increased—and hyperthyroid rats significantly decreased—ETP levels if compared to controls. High fat diet potentiated the ETP increasing effect in hypothyroid animals. Hence, the effect was so strong that every ETP value was far outside the highest of the other groups (Figure 1, filled downward directed triangles). Changes of fibrinogen levels followed the same pattern as ETP values (Table 1).

## 3. Discussion

Superficially viewed, these data support the hypothesis that hypothyreosis and increased fat intake synergistically generate a cardiovascularly unfavorable prothrombotic situation indicated by an increased ETP.

Viewed at a deeper level, however, individuals with active atherosclerosis, the main pathologic cause of cardiovascular disease (CVD), can benefit from such a constellation, if certain stages of their disease are taken into consideration. Most importantly, bleeding processes within atherosclerotic (AS) plaques are stabilized more effectively by an activated clotting system. Evidence for a major role of the clotting system in this context comes from a murine study in which it was shown that thrombin is centrally involved as a stabilizing factor. Hence, thrombin had pro-proliferative effects on arterial smooth muscle cells (SMCs), and influenced extracellular matrix synthesis in a positive way towards plaque stabilization [13]. Moreover, hypercoagulability reduced the transendothelial migration of monocytes into AS plaques [13]. A decrease of intraplaque bleeding and thus free heme accumulation will also decelerate the inflammatory activity of monocytes and foam cell activation of macrophages around the lipid core [14]. This is explained by the fact that the iron in heme is an effective peroxidant catalyst via H_2_O_2_ coordination and Fenton chemistry [15]. Hence, hemoglobin deposition enhances the lipid peroxidation of cholesterol. Admittedly, these data are from a murine AS model which have to be transferred with caution to the human situation [13]. Nevertheless, as shown recently in two human cohorts, cardiovascular patients with strongly elevated ETP levels had indeed a better overall prognosis [11,16]. Thus, the hypothesis suggests that a strong, well responsive, and alert clotting reactivity rather prevents high risk candidates from cardiovascular end points than causes clinical events by thromboembolic processes. Positive effects further comprise ubiquitous intravascular “sealing up” effects, probably most effective at the surface of AS plaques (fibrous cap).

Moreover, clinical effects of thyroid hormones may also play a role in this context which has been so far underestimated. Hence, latent hypothyreosis may stabilize bleeding processes in vulnerable plaques of obese people by switching the clotting system to an alert state. Accordingly, an increased ETP, as observed in our rat model together with hypothyreosis, may lower the incidence of fatal post-myocardial events. A new question arises as to whether to treat these patients by anticoagulation or not.

Regarding body weight changes in various groups it has to be mentioned that findings in rats cannot be transferred to patients with thyroid dysfunctions, as described in detail [17]. The main reason is the declined development and growth of hypothyroid rats, due to missing appropriate thyroid hormones for growth, and on the other hand the increased metabolic rate in hyperthyroid rats, which results in decreased body weight.

Furthermore, we found no significant lower total T3 levels in hypothyroid animals under high fat diet which seems that higher calorie intake in part stimulates thyroid hormone production as we also see an increase of T3 in control rats under high fat diet compared to normal fed controls.

Limitations: Thyroid hormone suppression in rats as a consequence of propylthiouracil treatment may induce side effects, e.g., hepatotoxicity. To avoid such side effects, dose and duration of treatment have been based on knowledge from previous studies where the treatment was well tolerated [17,18,19,20]. Furthermore, the normal antithrombin levels contradict liver damage. Nevertheless, our findings have to be proven in further studies, especially regarding the mechanistic background.

Taken together, we are the first to show that hypothyroidism and high fat diet potentiate the thrombotic capacity of the clotting system in Sprague Dawley rats. This may be relevant for cardiovascular diseases where thyroid function has been so far neglected so that an improved pathophysiological understanding of different risk phenotypes can be obtained.

## 4. Experimental Section

### 4.1. Animals

Female Sprague Dawley (SD) rats (Himberg, Austria), were housed (three per cage) and maintained on a 12 h light:12 h dark cycle in a temperature-(21 ± 2 °C) and humidity-controlled animal facility. The animals were acclimatized for one week under the same laboratory conditions of photoperiod, humidity and room-temperature. Rats initially weighing 251 ± 21 g were either assigned to normal rodent diet groups (Altromin, Lage, Germany) with 2.6 kcal/g and 11% fat or custom-designed high-fat diet (HFD) groups with 4.7 kcal/g and 56% fat (Harlan, Vienna, Austria). The HFD composition was based on previous studies [21,22,23]. Food and tap water were provided ad libitum. Experimental setup for female SD rats was performed according to the guidelines of the Animal Care and Use Committee of the Ministry of Science and Research, Vienna, Austria and approved by the responsible national ethics committee, Ministry of Science and Research, Vienna, Austria, ID 162-2010/11; October 2010.

### 4.2. Experimental Design and Treatment

The rats were randomly allocated into six groups as shown in Table 1. Groups 1–3 were fed with normal rodent diet, whereas groups 4–6 were fed with HFD. Hypothyroidism was induced via administration of 6-propylthiouracil (0.04 g/100 mL) into tap drinking water over the whole experimental period of 12 weeks as described [17,18,19]. Hyperthyroidism was induced with 3,3′,5-triiodothyronine (T3) (300 μg/kg in 0.50 mM NaOH) via intraperitoneal (i.p.) injections every other day for 12 weeks [18].

### 4.3. Blood Collection

After 12 weeks, blood was obtained via heart puncture after an overnight fast. Rats were anesthetized with isofluran (Forane, Abbott, Vienna, Austria) prior to blood sampling. Blood was collected using S-Monovette^®^ Serum-Gel tubes, Sarstedt, Vienna, Austria and centrifuged at ambient temperature. Samples were aliquoted and stored at −80 °C until analysis.

### 4.4. Laboratory Procedures

Platelet free plasma, derived from citrated blood samples was used for ETP analysis, which was performed by the new CE-IVD labeled Innovance^®^ ETP test kit on a BCS-XP analyzer (Siemens Healthcare Diagnostics, Marburg, Germany). Serum triiodothyronine (T3) was determined by commercial Rat ELISA (Uscn Life Science Inc., Wuhan, China). During the course of treatment, body weight measurements were performed at a 3-week interval and food consumption and calorie intake were recorded. The amount of ingested diet was calculated as the difference between the amount of food that was placed in the food bin and the weight of food that remained in the bin. Calorie intake per rat per day was calculated via food consumption per animal per day and the diets’ metabolizable energy values as kilocalories per gram (kcal/g). Total cholesterol, free cholesterol, and HDL cholesterol (homogeneous assay) were measured using enzymatic reagents from Diasys (Holzheim, Germany) and were calibrated using secondary standards from Roche Diagnostics (Mannheim, Germany). Free fatty acids were measured using enzymatic reagents form Wako Chemicals (Neuss, Germany). All measurements were performed on an Olympus AU640 (Producer, Vienna, Austria) automatic analyzer.

### 4.5. Statistical Analysis

Data are presented as means ± standard deviations. Continuous variables were compared using Students *t*-test for independent samples or Mann–Whitney *U*-test depending on the distribution of data. Correlations between variables were determined by linear regression analysis according to Pearson (r, Pearson correlation coefficient; P, univariate ANOVA). *p*-values < 0.05 were considered statistically significant. Analyses were performed by explorative data analyses using SPSS for Windows (SPSS Inc., Chicago, IL, USA).
